# Lichtenberg figures—morphological findings

**DOI:** 10.1007/s12024-023-00612-7

**Published:** 2023-04-12

**Authors:** Roger W. Byard

**Affiliations:** 1 Adelaide School of Biomedicine, The University of Adelaide, Level 2, Room N237, Helen Mayo North, Adelaide, 5005 SA Australia; 2 Forensic Science SA, Adelaide, Australia

**Keywords:** Lichtenberg figures, Ferning, Lightning, Capillary dilatation keraunopathology

## Abstract

Following a witnessed lethal lightning strike of an adult male who was standing outside in a storm, numerous Lichtenberg figures were identified upon external examination of the body. Sectioning across multiple areas of linear erythema in the figures showed no subcutaneous hemorrhage. This was later confirmed on histology which showed only subtle dermal capillary dilatation with no interstitial hemorrhage or inflammation in these areas. The only areas of interstitial hemorrhage were present in adjacent scattered punctate burns from arcing. The documented resolution of Lichtenberg figures within hours would be more in keeping with temporary functional capillary dilatation, shown in this case, rather than with tissue alteration by interstitial hemorrhage or inflammation.

## Case report

Two men who were standing outside watching an approaching thunderstorm were struck by lightning. One was rendered unconscious and the other immediately killed. The decedent was referred for medicolegal assessment which consisted of an external examination with skin sampling, CT examination, and toxicological evaluation of blood, all performed 3 days after death. There was no history of significant medical illness.

**Fig. 1:**
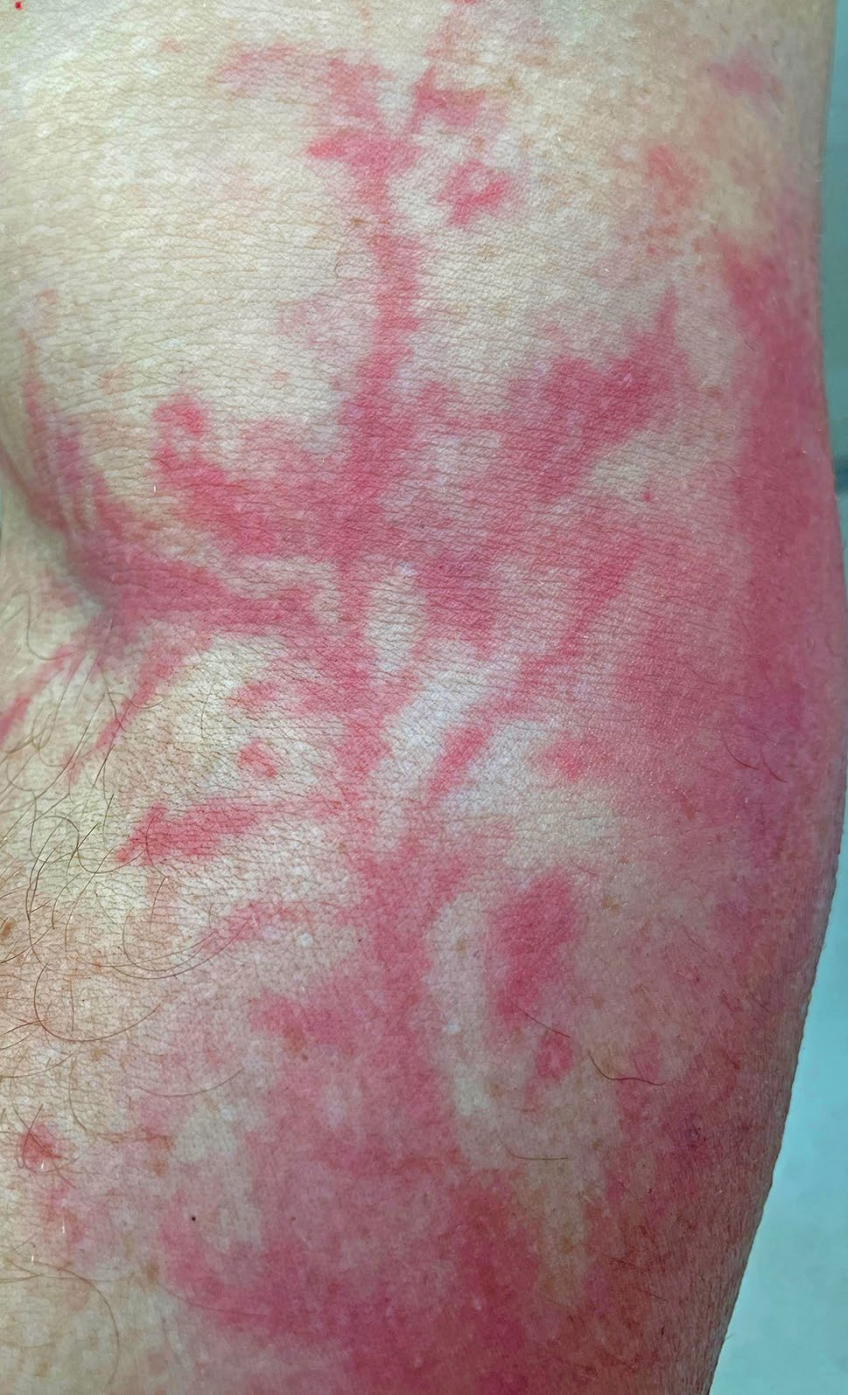
A Lichtenberg figure in the cubital fossa showing a typical arborizing pattern of erythema

**Fig. 2:**
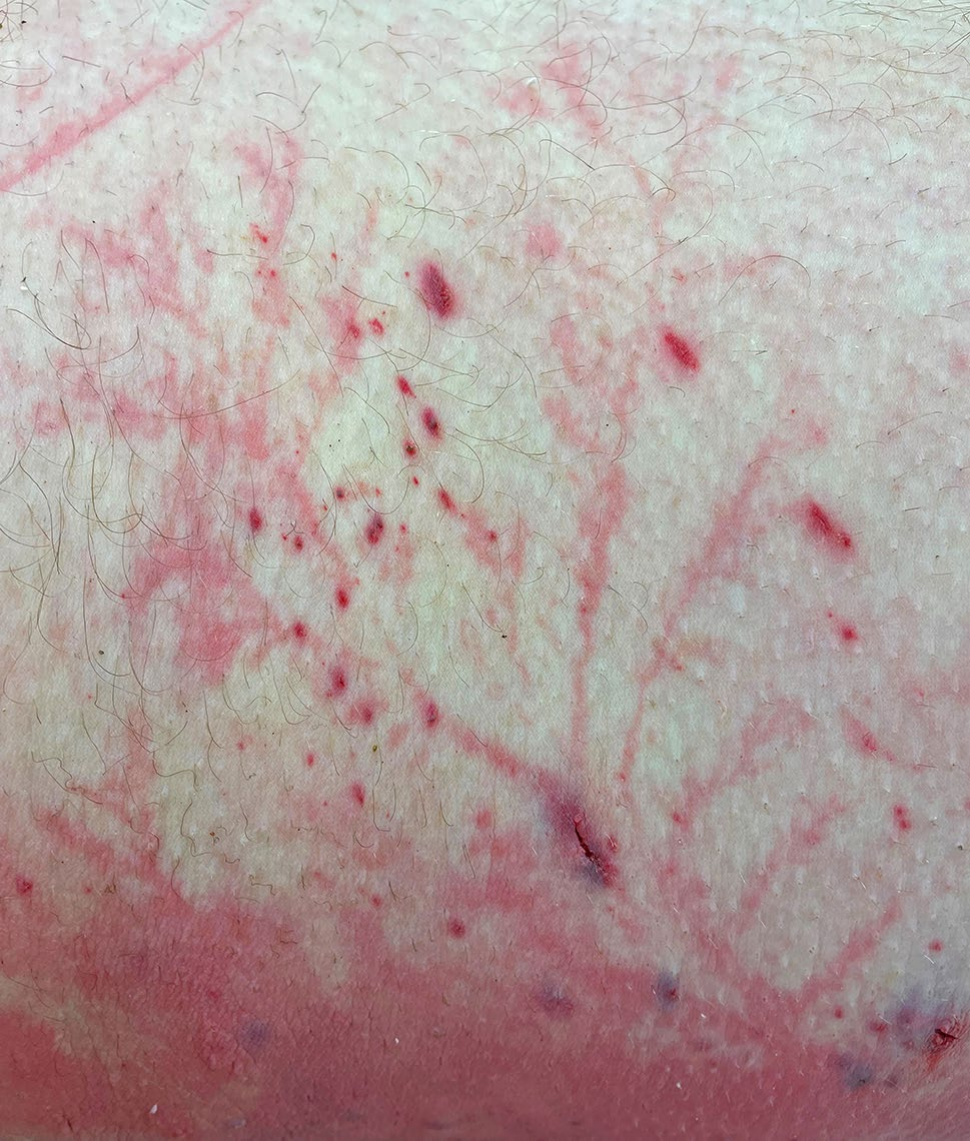
An area on the inner thigh showing similar although finer lines. Also present in this area and extending into adjacent skin where there were no Lichtenberg figures were numerous punctate burns associated with subcutaneous hemorrhage

At autopsy, the body was that of an adult male in his twenties. He had only been wearing a pair of underpants. The only signs of injury consisted of multiple Lichtenberg figures on the inner upper left thigh, the right cubital fossa (Fig. 1), the upper right flank, the upper anterior left flank, and the left forearm. There were also multiple small (1–2 mm) punctate arcing burns on the inside of both thighs (Fig. 2), the scrotum, and the lower abdomen. Incising of the figures revealed no interstitial hemorrhage, whereas incising of the punctate burns revealed clear evidence of dermal and subcutaneous bleeding.

**Fig. 3:**
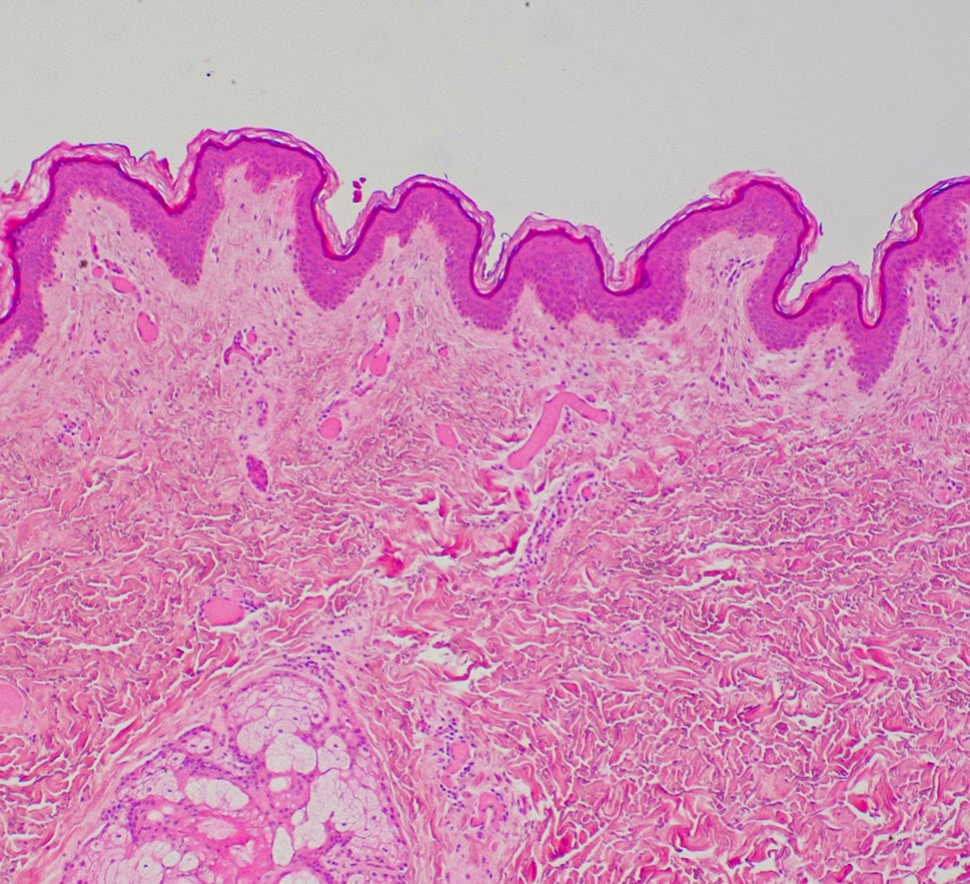
Histological examination of the areas of linear erythema in all of the Lichtenberg figures revealed a similar picture with no interstitial hemorrhage, inflammation or epidermal, dermal or subcutaneous injuries. The only findings were of subtle dermal capillary dilatation, contrasting with adjacent normal skin (hematoxylin & eosin, H&E × 100)

**Fig. 4:**
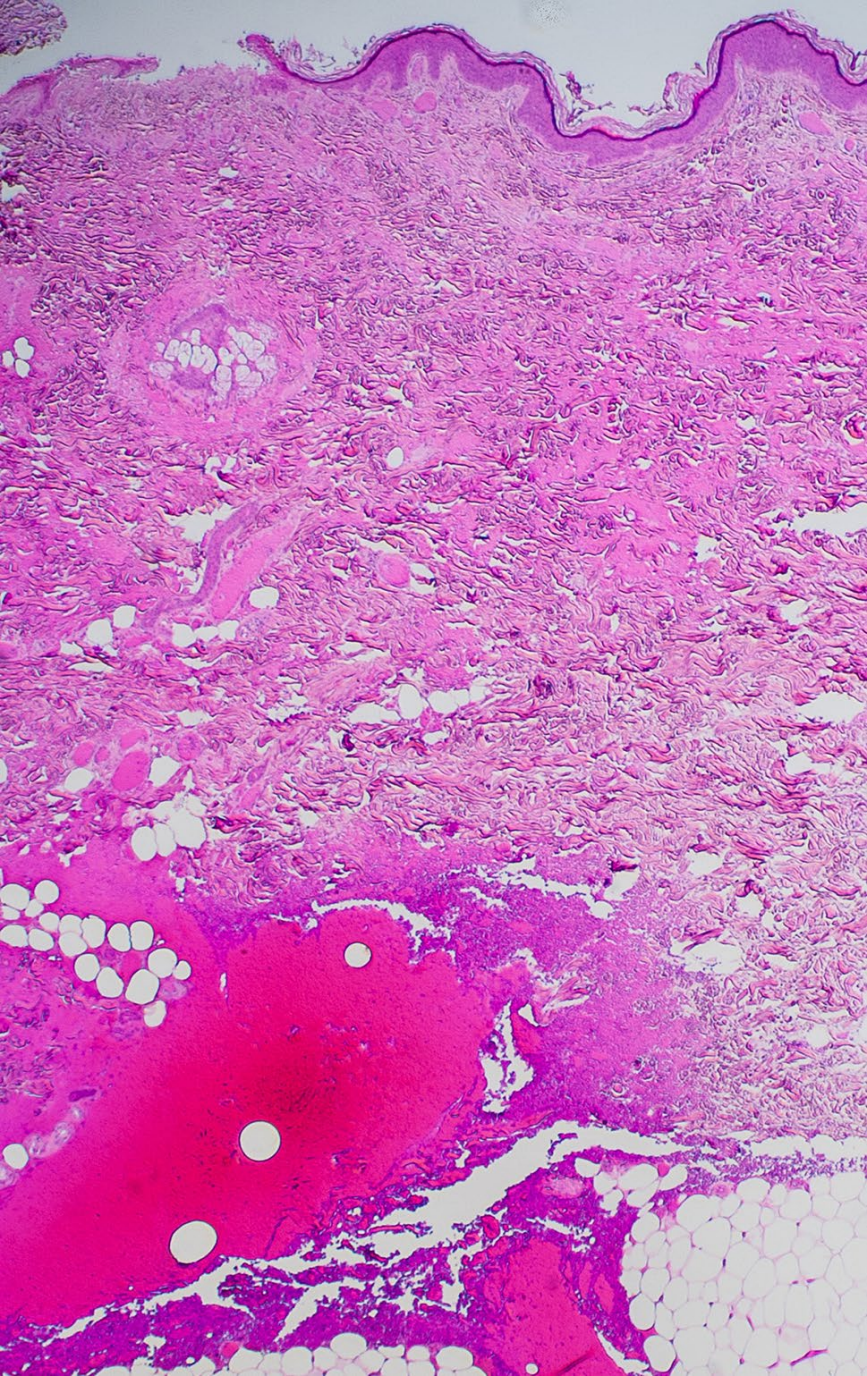
Prominent interstitial hemorrhage was present only in sections from areas of punctate burning from arcing. Superficial skin loss is noted (H&E × 100)

Histological examination of the Lichtenberg figures showed subtle dilatation of superficial dermal capillaries with no evidence of any associated interstitial hemorrhage or inflammation (Fig. 3). The only areas of dermal and subcutaneous hemorrhage (without inflammation) were found in the biopsies of the arc burns associated with loss of superficial epidermis and very mild nuclear streaming (Fig. 4). CT examination was unremarkable. Toxicological evaluation of blood revealed 0.095% alcohol and tetrahydrocannabinol with no other common drugs. Death was attributed to lightning strike.

## Discussion

Lichtenberg figures were first described by a German physicist in the late eighteenth century during experiments with static electricity [1]. Transient lines of erythema with a fern-like pattern that occur in lightning strikes are also referred to as Lichtenberg figures. These appear approximately 1 h after a strike and fade within hours in survivors [2–4]. They are not burns and do not follow nerve or vessel pathways and are not associated with damage to the epidermis or underlying tissues [5]. They have been reported in a survivor of a telephone lightning strike [6].

As there have been surprisingly few reports of the histological characteristics of these lesions and as there also appears to be some confusion in the literature regarding the microscopic morphology of these figures, the current case was reported.

Lightning occurs when a very rapid (0.0001–0.001 s) discharge of static electricity occurs during a thunderstorm. The current can be as high as 200,000 A with voltages between 20 million and 1 billion volts. It may occur in the absence of thunder. Skin lesions caused by a strike range from singeing of hair to extensive deep charring [7], although on occasion there may be no observable external effects. While Lichtenberg figures were once regarded as pathognomonic for lightning strike [2], they have been reported in a case of high-voltage industrial contact [8]. They are also present in only 17–33% of cases of lightning strike [9], but in the correct context of a suspected strike remain strongly supportive pathological evidence.

The etiology of Lichtenberg figures remains unclear with no theory adequately explaining their occurrence. While it has been suggested that they follow lines of skin moisture, their absence in the axillae and groin creases in favor of drier more exposed areas would not fit with this concept. Similarly the idea that they follow superficial vessels has been discounted [10]. Although the figures have been called “fractal burns,” there is usually no evidence of burning of the skin. The fractal concept is based on a mathematical model which suggests that the figures are a response to a positive electrical discharge [11].

There has also been some disagreement as to the precise morphology of these figures, with some authors attributing their appearance to interstitial hemorrhage [10] or to inflammation [6]. It is, however, unclear how interstitial aggregates of red blood cells or inflammatory cells could disperse so rapidly given that similar collections of such cells in bruises may take weeks to disappear [12]. As Resnick and Wetli noted, “their rapid nonscarring resolution remains a mystery” [10].

However, in the reported case, cutting across the lines of erythema showed no macroscopic evidence of hemorrhage, and in the histologic sections, the most characteristic finding was simple dermal vessel dilatation. If this is the underlying pathology, then the mystery of the rapid resolution of the figures in survivors could be readily explained, as dilated capillaries would certainly have the capacity to more rapidly revert to normal compared to extravascular aggregates of red cells, which require erythrocyte breakdown and removal by macrophages. For example, histologic examination of the linear areas of erythema in Fig. 1 from the cubital fossa merely revealed focal dermal capillary dilatation with no interstitial component (Fig. 3). In contrast, areas of interstitial hemorrhage were noted subjacent to punctate burns (Figs. 2 and 4) which not uncommonly accompany Lichtenberg figures. Thus, the histologic features reported previously with interstitial hemorrhage [9] may have been due to the additive effect of two distinct processes: arcing burns and Lichtenberg figures. The presence of inflammation [6] has not been confirmed.

In conclusion, Lichtenberg figures are a striking skin finding in a minority of cases of lightning strike that may be present in both the living and the dead. Their etiology is poorly understood; however, their rapid resolution in survivors would be more in keeping with temporary capillary dilatation rather than with interstitial hemorrhage or inflammation, as has been previously suggested.
